# Specific features to differentiate Giant cell arteritis aortitis from aortic atheroma using FDG-PET/CT

**DOI:** 10.1038/s41598-021-96923-2

**Published:** 2021-08-30

**Authors:** Olivier Espitia, Jérémy Schanus, Christian Agard, Françoise Kraeber-Bodéré, Jeanne Hersant, Jean-Michel Serfaty, Bastien Jamet

**Affiliations:** 1 Department of Internal and Vascular Medicine, CHU de Nantes, 1 place Alexis Ricordeau, 44093 Nantes, France; 2 Université de Nantes, Nantes, France; 3 Department of Nuclear Medicine, CHU de Nantes, 44000 Nantes, France; 4 Université de Nantes, CHU de Nantes, CNRS, Inserm, CRCINA, 44000 Nantes, France; 5 Department of Cardiovascular Imaging, CHU de Nantes, 44000 Nantes, France

**Keywords:** Rheumatic diseases, Diagnostic markers

## Abstract

Aortic wall ^18^F-fluorodeoxyglucose (FDG)-uptake does not allow differentiation of aortitis from atheroma, which is problematic in clinical practice for diagnosing large vessel vasculitis giant-cell arteritis (GCA) in elderly patients. The purpose of this study was to compare the FDG uptake characteristics of GCA aortitis and aortic atheroma using positron emission tomography/FDG computed tomography (FDG-PET/CT). This study compared FDG aortic uptake between patients with GCA aortitis and patients with aortic atheroma; previously defined by contrast enhanced CT. Visual grading according to standardized FDG-PET/CT interpretation criteria and semi-quantitative analyses (maximum standardized uptake value (SUV_max_), delta SUV (∆SUV), target to background ratios (TBR)) of FDG aortic uptake were conducted. The aorta was divided into 5 segments for analysis. 29 GCA aortitis and 66 aortic atheroma patients were included. A grade 3 FDG uptake of the aortic wall was identified for 23 (79.3%) GCA aortitis patients and none in the atheroma patient group (p < 0.0001); grade 2 FDG uptake was as common in both populations. Of the 29 aortitis patients, FDG uptake of all 5 aortic segments was positive for 21 of them (72.4%, p < 0.0001). FDG uptake of the supra-aortic trunk was identified for 24 aortitis (82.8%) and no atheromatous cases (p < 0.0001). All semi-quantitative analyses of FDG aortic wall uptake (SUV_max_, ∆SUV and TBRs) were significantly higher in the aortitis group. ∆SUV was the feature with the largest differential between aortitis and aortic atheroma. In this study, GCA aortitis could be distinguished from an aortic atheroma by the presence of an aortic wall FDG uptake grade 3, an FDG uptake of the 5 aortic segments, and FDG uptake of the peripheral arteries.

## Introduction

Giant cell arteritis (GCA) is the most frequent systemic vasculitis. Contrast enhanced computed tomography (CECT) and ^18^F-fluorodeoxyglucose positron emission tomography/computed tomography (FDG-PET/CT) are both currently recommended by the European league against rheumatism (EULAR)^1^ for large vessel assessment.

Whilst FDG-PET/CT enables visualization of vessel wall FDG uptake, this has not been specifically demonstrated for vasculitis. A meta-analysis demonstrated that the presence of vascular FDG uptake equal to or greater than liver background uptake on FDG-PET/CT was the best criterion for the detection of vascular inflammation in patients with GCA compared to controls (pooled sensitivity 90%, specificity 98%)^2^. However, there are difficulties in the use of FDG-PET/CT in GCA diagnosis with a lack of consensus on PET criteria to define thresholds of vascular inflammation in comparison to atheroma or healthy controls, as well as overestimation of FDG uptake from the vascular wall because of the presence of arteriosclerosis in these elderly^2^. Indeed, aortic atherosclerotic vascular uptake, more frequently observed in the aged population, may be a source of false positivity for evaluation of large vessel vasculitis (LVV) despite a classical “patchy” uptake pattern and a glucose uptake with lower intensity than LVV.

Recently, Stellingwerff et al.^3^ measured FDG uptake in GCA patients compared to a control calcified aortic population; however there was no baseline CECT to reliably classify the patients in this study. In addition, the atheromatous patient group was defined exclusively by the presence of wall calcifications while many atheromatous lesions are not calcified.

To our knowledge, no study has directly compared FDG uptake of the aortic wall according to the presence of LVV or atheroma with CECT as the reference. The aim of this retrospective study was to compare aortic wall FDG uptake between GCA aortitis and aortic atheroma, both preliminarily defined by aortic CECT, and to identify the best visual and semi-quantitative FDG-PET/CT features to discriminate aortitis from atheromatous aortic lesions.

## Methods

### GCA aortitis patients

This study included patients diagnosed with aortitis related to GCA between June 2014 and June 2021. Each GCA case included in this study had a CECT and a FDG-PET/CT before corticosteroid therapy or no more than 10 days after its initiation.

All GCA patients had to meet at least three American College of Rheumatology (ACR) criteria for the diagnosis of GCA^4^; or be over 50 years of age with elevated C-reactive Protein (CRP) and an aortitis identified by CECT.

**Figure 1:**
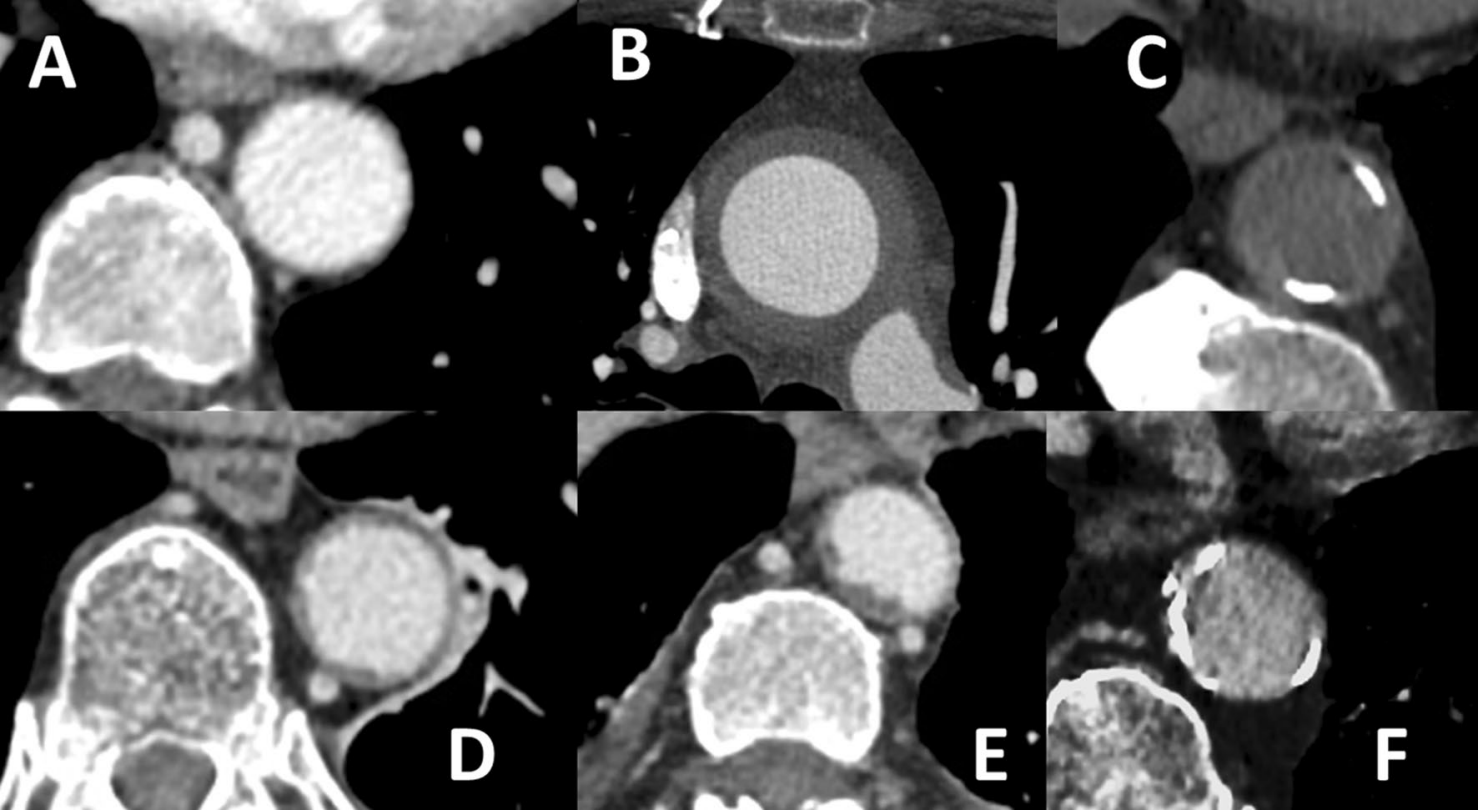
Aortic evaluation with and without contrast enhanced computed tomography (CT); (**A**) Normal aorta, (**B**) Aortitis, (**C**) Aortic calcification in non-enhanced CT, (**D**) Atheroma grade 1, (**E**) Atheroma grade 2, F: grade 2 mixed atheroma with calcification.

Aortitis was defined by CECT with a circumferential aortic parietal thickening > 2.2 mm^1^ (Fig. 1).

### Aortic atheroma control patients

Each GCA related aortitis patient was matched with 2 to 3 controls with proven CECT aortic atheroma. Controls with aortic atheroma had to have at least 2 out of 5 CT-positive aortic segments to be included in the study. Matching was done on both sex and age. The aortic atheromatous control patients were drawn from a group of patients with a history of neoplasia followed with FDG-PET/CT and CECT. The time between the 2 examinations was less than 3 weeks.

All control cases were free of neoplasia at the time of assessment and had not received oncology treatment for at least 3 months. Patients with active cancer or who had been treated within 3 months were excluded.

Aortic atheroma was defined by CT as an atheromatous lesion with a semi-quantitative ranging ≥ 1 (score ranging from 0 to 2: 0 for the absence of plaque; 1 for the presence of smooth thin plaques and 2 for the presence of thick irregular plaques (≥ 3 mm))^5^ (Fig. 1).

### FDG-PET/CT acquisition and analysis

PET acquisition method: after at least 6 h of fasting, 3 MBq/kg of ^18^F-FDG was injected intravenously (after recording baseline blood glucose level). After 60 min of resting, FDG-PET/CT imaging was recorded in a supine position from the skull to the base of the thighs with arms next to the body. Images were acquired on a Siemens Biograph mCT64. First, non-contrast CT acquisition was performed with a multislice spiral CT scan. Next, a PET acquisition of the same axial range was performed with the patient in the same position. PET data were reconstructed using the Ordinary-Poisson OSEM provided by the manufacturer. All data were corrected for attenuation, scatter and random coincidences. The reconstruction parameters were 3 iterations, 21 subsets and a Gaussian post-filtering of 2 mm FWHM. The voxel size used was 4 × 4 × 2 mm The time per bed step was adapted following a methodology we previously published^6^.

**Figure 2:**
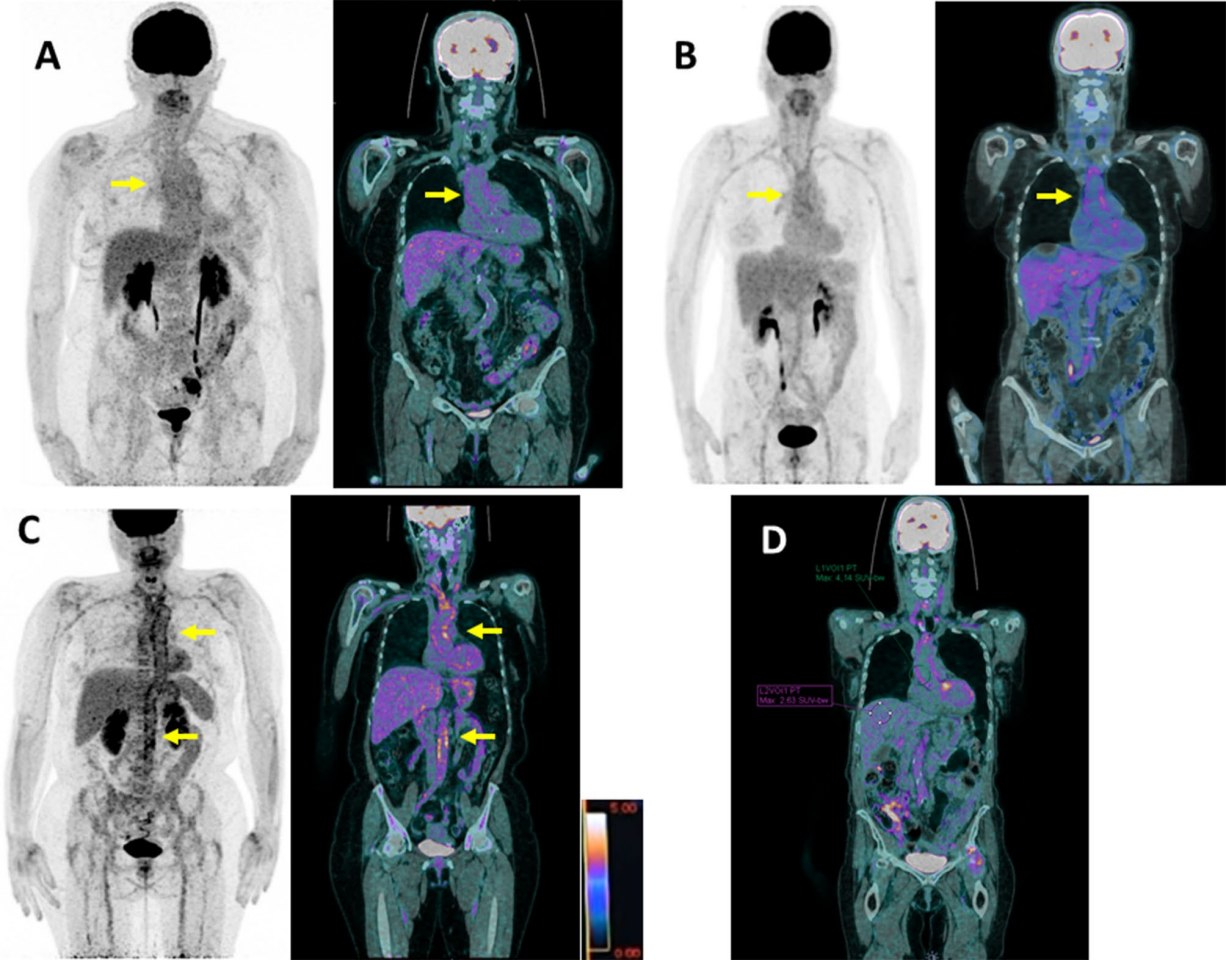
(**A**) FDG-PET/CT showing grade 1 uptake of the thoracic aortic wall (< liver uptake but > mediastinum background) in maximum intensity projection (MIP) image (left) and coronal fused (PET with CT) slice (right). SUV_max_ value of the thoracic aortic wall: 2.9; (**B**) FDG-PET/CT showing grade 2 (equal to liver background) large vessel vasculitis uptake (including the thoracic aorta) in maximum intensity projection (MIP) image (left) and coronal fused (PET with CT) slice (right). SUV_max_ value of the thoracic aortic wall: 4.2; (**C**) FDG-PET/CT showing grade 3 (> liver uptake) large vessel vasculitis uptake (including the thoracic and abdominal aorta) in maximum intensity projection (MIP) image (left) and coronal fused (PET with CT) slice (right). SUV_max_ value of the thoracic aortic wall: 6.9; (**D**) FDG-PET/CT coronal fused (PET with CT) slice illustrating the target-to-liver background ratio (TBR_max_ liver) in a patient with large vessel vasculitis. SUV_max_ value of the target (ascending thoracic aortic wall) is recorded with a region of interest (ROI) drawn manually around the target arterial structure. SUV_max_ value of the liver background is estimated with a ROI projected on the healthy right lobe to reduce the risk of including the various veins and arteries running through the liver. TBR_max_ liver of the ascending thoracic aorta: 4.14/2.63 = 1.57.

FDG-PET/CT was analyzed using a double blind centralized method, and in all cases the classifications were in agreement. A standardized grading system was used for FDG uptake visual analysis of the aorta and its main branches, with the background liver uptake used as the reference: grade 0, no significant vascular wall uptake (≤ mediastinum); grade 1, vascular wall uptake < liver uptake; grade 2, vascular wall uptake equal to liver uptake; grade 3, vascular wall uptake greater than liver uptake (Fig. 2)^7,8^.

**Figure 3:**
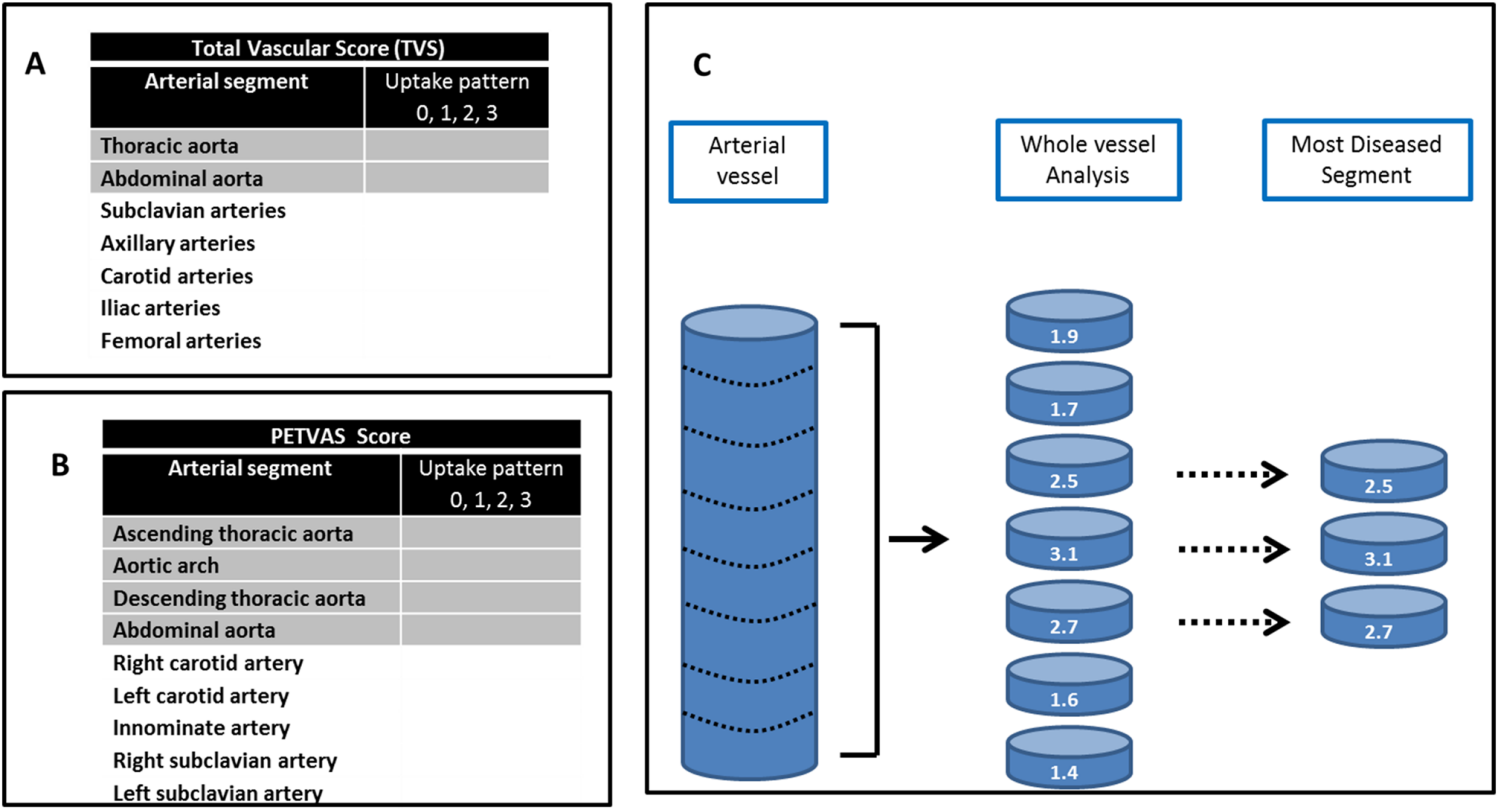
Total vascular score (**A**), PETVAS score, (**B**) and most diseased segment analysis (**C**). TVS and PETVAS are calculated by summing the highest FDG uptake pattern of each arterial segment presented in Table A and B.

Aortic images were segmented according to 5 anatomical regions: ascending thoracic aorta, aortic arch, descending thoracic aorta, abdominal suprarenal and infrarenal aorta. Total vascular (TVS)^9^ and PETVAS scores^10^ were performed (Fig. 3). An aortic score was calculated corresponding to the sum of the visual grading of the 5 aortic segments.

An analysis of the different aortic segments was performed by placing Regions of Interest (ROIs) around the vessel in cross section. The selected segments were defined according to the Most Diseased Segment (MDS)^11^, meaning that the slice with the highest SUV_max_ is selected and then the mean of the SUV_max_ from this and the two neighboring slices is calculated (Fig. 3).

Different target to background ratios (TBRs) were also recorded by measuring the maximal standardized uptake value (SUV_max_) of each reference organ. Ratios between aortic wall SUV_max_ and reference site SUV_max_ were evaluated by placing ROIs of similar size (4 cm^3^):

- Target-to-liver ratio (TBR_max_ liver) by placing a ROI on the right lobe of the liver (Fig. 2);
- Target-to-spleen ratio (TBR_max_ spleen) by placing a ROI on the spleen;
- Target-to-lung ratio (TBR_max_ lung) by placing a ROI at the level of a low activity area;
- Target-to-blood pool ratio (TBR_max_ blood) defined for supra-diaphragmatic vessels by a ROI drawn centrally in the blood pool of the superior vena cava and for infra-diaphragmatic vessels in the blood pool of the inferior vena cava;
- The delta (∆) SUV_max_ was also calculated as the difference between the aortic wall SUV_max_ and the venous SUV_max_.

Each of these parameters was compared between the aortitis and control group with aortic atheroma.

### CECT acquisition and analysis

CECT acquisition method: For the GCA aortitis patients, multi-slice spiral CECT scanning was performed with ECG triggering (to avoid motion or pulsation artifacts of the ascending aorta) with 120 kV, and the mAs range determined by automatic modulation dose and reconstruction slice thicknesses of 1 mm. First, non-contrast CT acquisition was performed, and then a non-ionic contrast agent (dose depending on the patient's body weight, body mass index, and kidney function) was injected through a venous catheter using an automated contrast material injector. Early arterial then portal-venous scan phases were acquired. For aortic atheroma control patients, only the portal-venous scan phase was recorded.

CECT was analyzed using a double blind centralized method. The aorta was divided into five segments and analyzed using the MDS method. For each segment, the following parameters were measured:

- Wall thickness: Maximal wall thickness was measured for the thickest part of the aorta. At least five measurements were made around the arterial wall and the mean was recorded as the maximal wall thickness of the corresponding aortic segment. These measurements were then performed away from any atheromatous lesions, identified by their irregular hypo intense appearance^5^;
- Aortic atheroma: Atheromatous lesions were assessed semi-quantitatively ranging from 0 to 2^5^. For each segment, the highest score was retained;
- Aortic diameter: This included the thickness of the arterial wall (outer circumference);
- The Muto ratio^12^ was measured (ratio between aortic wall thickness and external aortic diameter).
- Aortic calcifications were defined by a spontaneous density ≥ 130 UH. The quantification of calcifications was performed semi-quantitatively using the calcification thickness score method described by Tatsumi et al.^13^.

### Ethics

This study received ethics approval by the local board of ethics of the Nantes University Hospital. Each patient included in this study received written information and informed consent was obtained from all the participants. This research study was in accordance with the Declaration of Helsinski.

### Statistical analysis

Categorical variables were expressed in terms of counts and percentages, and quantitative variables were presented as means ± standard deviations (SD). The quantitative comparisons were assessed using a student’s t-test. Frequency comparisons were performed using Chi2 or Fisher’s exact test according to the statistical headcount. For all statistical analyses, a two-tailed p < 0.05 was considered significant. IBM SPSS Statistics v26 software was used for statistical analyses (Armonk, NY; USA).

## Results

### Patients

This study included 29 GCA patients with aortitis and 66 matched aortic atheroma controls. Mean age was 67.8 (± 9.3) and 70.9 (± 8.9) years respectively (p = 0.13). The number of women were 23 (79.3%) and 58 (87.9%) (p = 0.28) in the GCA aortitis group and the aortic atheroma group, respectively.

The cardiovascular risk factors were respectively for the aortitis group and the atheromatous group: hypertension 11 (37.9%) vs 45 (68.2%) (p = 0.006), diabetes 4 (13.8%) vs 8 (12.1%) (p > 0.99), dyslipidemia 9 (31.0%) vs 23 (34.8%) (p = 0.72), smoking history 9 (31.0%) vs 26 (39.4%) (p = 0.44), obesity 4 (13.8%) vs 22 (33.3%) (p = 0.08), history of stroke 1 (3.4%) vs 7 (10.6%) (p = 0.43), history of ischemic heart disease 3 (10.3%) vs 10 (15.2%) (p = 0.75) and history of arterial claudication or arterial ulcers of the lower limbs 2 (6.9%) vs 5 (7.6%) (p > 0.99).

In aortic atheroma group, 89.4% of patients had atheromatous lesions in at least 4 of the 5 aortic segments.

In the aortic atheroma control group, atheromatous patients had a history of neoplasia: 23 (34.8%) with hematologic malignancies, 17 (25.7%) with pulmonary neoplasia, 15 (22.7%) with melanomas, 7 (10.7%) with gastrointestinal cancers and 4 (6%) with other neoplasias (breast cancer, ENT, skin squamous cell carcinoma).

### FDG-PET/CT uptake patterns

Global FDG-PET/CT acquisition data are summarized in Table 1. In the GCA aortitis group, 11 (37.9%) patients had corticosteroids before FDG-PET/CT imaging. For these cases, the mean time between starting corticosteroids and FDG-PET/CT imaging was 3.7 days ± 3.2. The mean CRP value was 82.8 mg/l ± 64 when performing FDG-PET/CT imaging of the aortitis group.

**Table 1:** FDG-PET/CT uptake patterns global comparison between aortitis and aortic atheroma groups.

	GCA aortitis n = 29	Aortic atheroma n = 66	p
Mean 18FDG dose (MBq/kg) (± SD)	3.0 (± 0.1)	3.0 (± 0.1)	0.06
Mean time between injection and imaging (min) (± SD)	64.5 (± 7)	63 (± 7.9)	0.64
Mean Blood glucose level (mmol/l) (± SD) before imaging	5.2 (± 1.0)	5.4 (± 1.0)	0.35
Patients with liver enzyme disturbance n (%)	0 (0)	6 (9.1)	0.09
**Aortic FDG uptake:**			
Grade 3 aortic uptake n (%)	23 (79.3)	0 (0)	< 0.0001
**Grade 2 and/or 3 aortic uptake: n (%)**			
Uptake in 1 aortic segment	1 (3.4)	1 (1.5)	0.52
Uptake in 2 aortic segment	1 (3.4)	1 (1.5)	0.52
Uptake in 3 aortic segment	4 (13.8)	7 (10.6)	0.73
Uptake in 4 aortic segment	2 (6.9)	5 (7.5)	> 0.99
Uptake in 5 aortic segment	21 (72.4)	0 (0)	< 0.0001
Mean aortic score (± SD)	12.8 (± 2,9)	5.1 (± 2,0)	< 0.0001
Mean Total Vascular Score (± SD)	13.9 (± 4.0)	6.7 (± 1.5)	< 0.0001
Mean PETVAS score (± SD)	17.3 (± 4.1)	7.5 (± 2.2)	< 0.0001
**FDG peripheral arterial uptake**			
Supra-diaphragmatic vessels n (%)	24 (82.8)	0 (0)	< 0.0001
Sub-diaphragmatic vessels n (%)	16 (55.2)	6 (9)	< 0.0001

The global comparison of FDG-PET/CT uptake patterns between aortitis and aortic atheroma groups are summarized in Table 2.

**Table 2:** Comparison of FDG-PET/CT and CTA features in each group by aortic segment.

	GCA aortitis n = 29	Aortic atheroma n = 66	p
**Ascending thoracic aorta**			
**Visual aortic grading n(%)**			
Grade 0	0 (0%)	5 (7.6%)	0.32
Grade 1	3 (10.3%)	52 (78.8%)	< 0.0001
Grade 2	7 (24.1%)	9 (13.6%)	0.21
Grade 3	19 (65.6%)	0 (0%)	< 0.0001
**Quantitative **^**18**^**FDG uptake analysis** mean (± SD)			
SUV_max_	4.6 (± 1.5)	2.7 (± 0.4)	< 0.0001
∆SUV	2.8 (± 1.5)	0.9 (± 0.4)	< 0.0001
TBR_max_ blood	2.6 (± 1.0)	1.5 (± 0.3)	< 0.0001
TBR_max_ liver	1.4 (± 0.5)	0.8 (± 0.1)	< 0.0001
TBR_max_ spleen	1.7 (± 0.5)	1.1 (± 0.2)	< 0.0001
TBR_max_ lung	8.5 (± 3.3)	6.3 (± 1.8)	< 0.0001
**Aortic CT analysis**			
Mean aortic diameter (mm) (± SD)	36.0 (± 5.1)	34.7 (± 3.3)	0.15
Mean aortic wall thickening (mm) (± SD)	3.0 (± 1.6)	1.5 (± 0.3)	< 0.0001
Mean Muto ratio (± SD)	16.5 (± 6.1)	8.6 (± 2.2)	< 0.0001
Aortic atheroma semi-quantitative ranging ≥ 1 n (%)	2 (6.9%)	3 (4.5%)	0.64
Mean aortic calcification score (± SD)	0.4 (± 0.6)	0.4 (± 0.6)	0.82
**Aorta arch**			
**Visual aortic grading n(%)**			
Grade 0	0 (0%)	4 (6.1%)	0.31
Grade 1	2 (6.9%)	48 (72.7%)	< 0.0001
Grade 2	8 (27.6%)	14 (21.2%)	0.80
Grade 3	19 (65.6%)	0 (0%)	< 0.0001
**Quantitative **^**18**^**FDG uptake analysis mean (± SD)**			
SUV_max_	4.3 (± 1.5)	2.7 (± 0.5)	< 0.0001
∆SUV	2.5 (± 1.5)	0.9 (± 0.4)	< 0.0001
TBR_max_ blood	2.5 (± 1.0)	1.6 (± 0.3)	< 0.0001
TBR_max_ liver	1.4 (± 0.5)	0.9 (± 0.1)	< 0.0001
TBR_max_ spleen	1.6 (± 0.5)	1.1 (± 0.2)	< 0.0001
TBR_max_ lung	7.8 (± 2.7)	6.5 (± 1.8)	0.006
**Aortic CT analysis**			
Mean aortic diameter (mm) (± SD)	30.3 (± 4.2)	27.8 (± 2.9)	< 0.0001
Mean aortic wall thickening (mm) (± SD)	2.6 (± 1.2)	1.5 (± 0.3)	< 0.0001
Mean Muto ratio (± SD)	17.1 (± 5.1)	10.7 (± 2.4)	< 0.0001
Aortic atheroma semi-quantitative ranging ≥ 1 n (%)	7 (24.1%)	59 (89.4%)	< 0.0001
Mean aortic calcification score (± SD)	0.9 (± 0.9)	1.5 (± 0.6)	0.0003
**Descending thoracic aorta**			
**Visual aortic grading n(%)**			
Grade 0	0 (0%)	5 (7.6%)	0.32
Grade 1	1 (3.4%)	48 (72.7%)	< 0.0001
Grade 2	7 (24.1%)	13 (19.7%)	0.62
Grade 3	21 (72.4%)	0 (0%)	< 0.0001
**Quantitative **^**18**^**FDG uptake analysis mean (± SD)**			
SUV_max_	4.8 (± 1.7)	2.7 (± 0.5)	< 0.0001
∆SUV	3.0 (± 1.7)	0.9 (± 0.4)	< 0.0001
TBR_max_ blood	2.7 (± 1.3)	1.5 (± 0.3)	< 0.0001
TBR_max_ liver	1.5 (± 0.6)	0.8 (± 0.1)	< 0.0001
TBR_max_ spleen	1.8 (± 0.7)	1.1 (± 0.2)	< 0.0001
TBR_max_ lung	8.7 (± 3.7)	6.5 (± 1.9)	0.0003
**Aortic CT analysis**			
Mean aortic diameter (mm) (± SD)	29.8 (± 3.3)	26.1 (± 3.0)	< 0.0001
Mean aortic wall thickening (mm) (± SD)	3.2 (± 1.1)	1.4 (± 0.3)	< 0.0001
Mean Muto ratio (± SD)	21.1 (± 6.2)	10.8 (± 2.3)	< 0.0001
Aortic atheroma semi-quantitative ranging ≥ 1 n (%)	5 (17.2%)	65 (98.5%)	< 0.0001
Mean aortic calcification score (± SD)	0.6 (± 0.7)	1.0 (± 0.6)	0.003
**Supra renal abdominal aorta**			
**Visual aortic grading n(%)**			
Grade 0	2 (6.9%)	12 (18.2%)	0.21
Grade 1	2 (6.9%)	47 (71.2%)	< 0.0001
Grade 2	6 (20.7%)	7 (10.6%)	0.19
Grade 3	19 (65.6%)	0 (0%)	< 0.0001
**Quantitative **^**18**^**FDG uptake analysis mean (± SD)**			
SUV_max_	4.9 (± 2.1)	2.7 (± 0.5)	< 0.0001
∆SUV	3.0 (± 2.1)	0.8 (± 0.4)	< 0.0001
TBR_max_ blood	2.7 (± 1.3)	1.4 (± 0.2)	< 0.0001
TBR_max_ liver	1.5 (± 0.7)	0.8 (± 0.1)	< 0.0001
TBR_max_ spleen	1.8 (± 0.7)	1.1 (± 0.2)	< 0.0001
TBR_max_ lung	8.9 (± 4.2)	6.4 (± 1.7)	< 0.0001
**Aortic CT analysis**			
Mean aortic diameter (mm) (± SD)	26.1 (± 2.8)	22.6 (± 3.4)	< 0.0001
Mean aortic wall thickening (mm) (± SD)	3.0 (± 1.2)	135 (± 0.3)	< 0.0001
Mean Muto ratio (± SD)	22.8 (± 8.9)	11.4 (± 2.5)	< 0.0001
Aortic atheroma semi-quantitative ranging ≥ 1 n (%)	10 (34.5%)	63 (95.5%)	< 0.0001
Mean aortic calcification score (± SD)	0.8 (± 0.7)	1.2 (± 0.6)	0.03
**Infra renal abdominal aorta**			
**Visual aortic grading n(%)**			
Grade 0	2 (6.9%)	14 (21.2%)	0.07
Grade 1	2 (6.9%)	51 (77.3%)	< 0.0001
Grade 2	5 (17.2%)	1 (1.5%)	0.009
Grade 3	20 (69.0%)	0 (0%)	< 0.0001
**Quantitative **^**18**^**FDG uptake analysis mean (± SD)**			
SUV_max_	4.2 (± 1.4)	2.6 (± 0.5)	< 0.0001
∆SUV	2.3 (± 1.3)	0.7 (± 0.3)	< 0.0001
TBR_max_ blood	2.2 (± 0.7)	1.4 (± 0.2)	< 0.0001
TBR_max_ liver	1.3 (± 0.4)	0.8 (± 0.1)	< 0.0001
TBR_max_ spleen	1.5 (± 0.4)	1.0 (± 0.2)	< 0.0001
TBR_max_ lung	7.6 (± 2.8)	6.1 (± 1.7)	0.002
**Aortic CT analysis**			
Mean aortic diameter (mm) (± SD)	20.6 (± 2.6)	18.5 (± 2.4)	0.001
Mean aortic wall thickening (mm) (± SD)	2.7 (± 1.0)	1.2 (± 0.3)	< 0.0001
Mean Muto ratio (± SD)	26.2 (± 8.2)	12.7 (± 3.1)	< 0.0001
Aortic atheroma semi-quantitative ranging ≥ 1 n (%)	12 (41.4%)	62 (93.9%)	< 0.0001
Mean aortic calcification score (± SD)	1.0 (± 0.7)	1.4 (± 0.5)	0.02

*SUV* standardized uptake value, *TBR* target to background ratio, *∆SUV* delta standardized uptake value.

Grade 3 FDG uptake was only found in the aortic group. There was no difference in the frequency of grade 2 FDG uptake between the thoracic aorta and the supra-renal abdominal aorta groups (Table 2). Patients with aortic atheroma had significantly higher grade 1 FDG uptake in each aortic segment compared to patients with aortitis (Table 2).

Uptake higher or equal to grade 2 was found in all of the 5 aortic segments in 72.4% of aortitis patients, and none of the aortic atheroma patients (Table 1).

Comparisons of FDG-PET/CT uptake patterns in the aortitis group with or without corticosteroid therapy are summarized in Table 3.

**Table 3:** FDG-PET/CT uptake patterns comparison between aortitis cases with or without corticosteroid therapy before PET/CT.

	GCA aortitis without corticosteroid n = 18	GCA aortitis with corticosteroid n = 11	p
**Ascending thoracic aorta**			
**Visual aortic gradingn (%)**			
Grade 3	14 (77.8%)	5 (45.5%)	0.11
Grade 2	3 (16.7%)	4 (36.4%)	0.37
Grade 2 or 3	17 (94.4%)	9 (81.8%)	0.28
**Quantitative **^**18**^**FDG uptake analysis mean (± SD)**			
SUV_max_	5.1 (± 1.6)	3.8 (± 1.0)	0.03
TBR_max_ blood	2.9 (± 1.1)	2.1 (± 0.6)	0.06
TBR_max_ liver	1.6 (± 0.6)	1.2 (± 0.4)	0.06
**Aorta arch**			
**Visual aortic grading n(%)**			
Grade 3	14 (77.8%)	5 (45.5%)	0.11
Grade 2	3 (16.7%)	5 (45.5%)	0.20
Grade 2 or 3	17 (94.4%)	10 (81.8%)	> 0.99
**Quantitative **^**18**^**FDG uptake analysis mean (± SD)**			
SUV_max_	4.8 (± 1.7)	3.6 (± 0.5)	0.04
TBR_max_ blood	2.7 (± 1.1)	2.0 (± 0.4)	0.06
TBR_max_ liver	1.5 (± 0.5)	1.1 (± 0.2)	0.06
**Descending thoracic aorta**			
**Visual aortic grading n (%)**			
Grade 3	14 (77.8%)	7 (63.6%)	0.43
Grade 2	3 (16.7%)	4 (36.4%)	0.37
Grade 2 or 3	17 (94.4%)	11 (100%)	> 0.99
**Quantitative **^**18**^**FDG uptake analysismean (± SD)**			
SUV_max_	5.2 (± 1.9)	4.1 (± 0.9)	0.10
TBR_max_ blood	3.0 (± 1.5)	2.3 (± 0.5)	0.12
TBR_max_ liver	1.6 (± 0.7)	1.3 (± 0.2)	0.16
**Supra renal abdominal aorta**			
**Visual aortic grading n(%)**			
Grade 3	14 (77.8%)	5 (45.5%)	0.11
Grade 2	3 (16.7%)	3 (27.3%)	0.65
Grade 2 or 3	17 (94.4%)	8 (72.7%)	0.14
**Quantitative **^**18**^**FDG uptake analysis mean (± SD)**			
SUV_max_	5.5 (± 2.3)	3.9 (± 1.2)	0.06
TBR_max_ blood	3.0 (± 1.5)	2.1 (± 0.6)	0.07
TBR_max_ liver	1.7 (± 0.7)	1.2 (± 0.3)	0.09
**Infra renal abdominal aorta**			
**Visual aortic grading n(%)**			
Grade 3	15 (83.3%)	5 (45.5%)	0.05
Grade 2	2 (11.1%)	3 (27.3%)	0.34
Grade 2 or 3	17 (94.4%)	8 (72.7%)	0.14
**Quantitative **^**18**^**FDG uptake analysis mean (± SD)**			
SUV_max_	4.5 (± 1.4)	3.6 (± 1.2)	0.11
TBR_max_ blood	2.4 (± 0.7)	2.0 (± 0.7)	0.12
TBR_max_ liver	1.4 (± 0.4)	1.1 (± 0.4)	0.11

### FDG-PET/CT quantitative analysis and CECT analysis

Each quantitative feature reflecting FDG uptake was significantly different between the two groups (Table 2). Mean SUV_max_ and ∆SUV ranged from 4.2 to 4.9 and 2.5 to 3.0 (according to the aortic segment) in the aortitis group and from 2.6 to 2.7 and 0.7 to 0.9, respectively, in the aortic atheroma group.

The most discriminative FDG-PET/CT quantitative features between the two groups were ∆SUV and TBR blood. Depending on the aortic segment, mean ∆SUV was 2.8 to 3.8 times greater in the aortitis group compared to the aortic atheroma group. Moreover, all mean TBR_max_ ratios were significantly higher in the aortitis group.

For each aortic segment, CECT analysis showed significantly higher aortic diameters, higher aortic wall thickness and higher Muto ratios in the aortitis group. Nine (31%) patients with aortitis had atheroma in the thoracic aorta and 12 (41.4%) in the abdominal aorta (Table 2).

## Discussion

To our knowledge, this is the first study that directly compares FDG-PET/CT uptake patterns of the aortic wall in GCA aortitis and aortic atheroma patients all previously defined by CECT.

This study showed that in daily practice, if a patient suspected of GCA presents a grade 3 FDG uptake of the aorta by FDG-PET/CT, FDG uptake involving the 5 aortic segments, and if in addition to the aortic FDG uptake there is FDG uptake within the supra-aortic trunks, the diagnosis of GCA associated with large vessel vasculitis is very likely, even in patients with many cardiovascular risk factors and atheroma. Moreover, the global analysis found significantly higher TVS and PETVAS scores in the aortitis group where an extensive FDG uptake was more frequently associated with involvement of both supra and sub-diaphragmatic large vessels.

This study showed that grade 2 FDG uptake was as frequent for GCA aortitis and aortic atheroma patients. In this situation, where a definitive diagnosis using FDG-PET/CT is not possible, additional CECT imaging should be considered to distinguish aortitis from atheromatous plaques.

By semi-quantitative analysis, SUV_max_, ∆SUV and the different TBRs, regardless of organ references and/or aortic segment, were significantly higher in the aortitis group. ∆SUV, which is to our knowledge for the first time assessed in a FDG-PET/CT vasculitis study, was the most discriminative parameter between aortitis and aortic atheroma^14^.

There are different methods to analyze FDG uptake patterns in LVV. Visual grading system analysis (with liver background uptake as the reference) has the highest interobserver agreement (kappa: 0.96 in initial study and 0.79 in external validation)^15^ when vascular wall FDG uptake higher than liver uptake is used as a diagnostic criterion, although agreement is also good without predefined criteria (kappa: 0.68 and 0.85). Sensitivity and specificity are comparable with these methods. The criterion of vascular wall FDG uptake equal to liver FDG uptake has low specificity^15^. This study confirmed that compared to the FDG uptake of aortic atheroma, an aortic uptake superior to the liver was exclusively found in the aortitis group. FDG aortic wall uptake equal to the liver background was as frequent in the aortitis as in the atheroma group.

The use of ∆SUV or TBR instead of SUV raw values may reduce the effects of signal quantification errors due to patient weight, injected radiotracer dose and imaging time point. However, standardized thresholds for distinguishing aortitis, atheroma and normal values need to be further explored and defined^3,16^.

This study has several limitations, such as the number of patients included and the maximum interval of 10 days between the start of corticosteroid therapy and FDG-PET/CT imaging. FDG uptake and consequently the test’s sensitivity decreases significantly after glucocorticoid exposure^17^. Although the intensity of vascular FDG uptake in GCA declines with glucocorticoid treatment, long-term persistent vascular FDG uptake may be present despite clinical remission^18^. In this study, only 37.9% of patients with aortitis had corticosteroids prior to FDG-PET/CT imaging due to the risk of ophthalmological complications. For those who had corticosteroids, exposure was short (mean 3.7 days). However, each included patient had an aortitis on the CECT scan and several studies have demonstrated a correlation between the two examinations. Moreover, FDG-PET/CT scans have been performed with analogical devices so it is possible that the results of this study (especially the quantitative analysis results) would have been slightly different if new generation digital PET/CT devices had been used. Indeed, these new devices offer better spatial resolution reducing the partial volume effect (PVE) and could improve FDG uptake signals for small atheromatous plaques. The PVE probably reduced the signal measured from small atherosclerotic plaques that is unavoidable. However, PVE depends on the surrounding signal too. Moreover, aortic wall SUV_max_ is also probably influenced by the PVE in aortitis as this is usually a thin (about 3 mm) structure. Thus, the aortic wall thickness in aortitis and in aortic atheroma is very similar, therefore limiting the risk of bias.

## Conclusion

This study is the first that directly compares FDG-PET/CT uptake patterns of the aortic wall in GCA aortitis and aortic atheroma patients, all previously defined using CECT. A grade 3 FDG uptake by visual analysis of the aortic wall was only identified in patients with aortitis. A grade 2 FDG uptake was as common in aortitis as in aortic atheroma patients. FDG uptake of the 5 aortic segments was exclusively found in aortitis patients and FDG uptake of the supra-aortic trunks was found in 82.8% of aortitis patients (never in atheromatous cases). All quantitative FDG-PET/CT features of the aortic wall (SUV_max_, ∆SUV and TBRs) were significantly higher in the aortitis group. ∆SUV was the most discriminative feature between aortitis and aortic atheroma. Larger prospective studies are required to validate the results of this study and to define diagnostic thresholds for aortitis.
